# What best animal science teachers do

**DOI:** 10.1093/tas/txaa223

**Published:** 2020-11-28

**Authors:** B D Whitaker, W R Lamberson, M F Smith, B S Walters, C R Youngs

**Affiliations:** 1 Department of Animal and Pre-veterinary Studies, University of Findlay, Findlay, OH, USA; 2 Division of Animal Sciences, University of Missouri, Columbia, MO, USA; 3 Department of Animal and Food Science, University of Wisconsin River Falls, River Falls, WI, USA; 4 Department of Animal Science, Iowa State University, Ames, IA, USA

**Keywords:** classroom, educators, pedagogy, teach, undergraduate education

## Abstract

Great teachers have the extraordinary ability to inspire and motivate even those students who resist learning. The top educators are knowledgeable not only about the content of the course they are teaching but also of the information, literature, and practice of instructional delivery to their audience. Many exemplary educators have been profiled and studied; however, there is a paucity of information pertaining to how the top animal science teachers teach. The objective of this study was to identify and describe characteristics of award-winning animal science teachers. The inclusion criterion for selecting faculty was being bestowed an excellence in teaching award through their professional organization. Each teacher answered a series of questions about themselves, their students, and the class being taught. Lecture was captured using a digital all-inclusive camera and later analyzed for pedagogical trends and instructor–student interactions. Despite a variety of topics being taught by award-winning teachers, there were multiple trends emerging from their classrooms. Common events included reviewing highlights of previous lectures, distributing something to students, posing questions during class, and calling on students by name. Each teacher taught differently, but they all understood their audience; they grasped the subject matter and most importantly, they valued students learning. Collectively, these findings can be utilized and applied by animal science teachers in their own environments in an attempt to foster improved student learning through excellent teaching.

## INTRODUCTION

When *What the Best College Teachers Do* (Bain, 2004) was published, it arguably reinvigorated the teaching pedological landscape in higher education. This book articulated that the best teachers facilitate learning that makes sustained, substantial, and positive influences in students’ development. His research and observations of over 60 teachers at a variety of institutions suggested that their students were satisfied with the instruction they received and had a desire to continue learning. Teachers encouraged their students to learn in ways that gained respect from their colleagues and the community. More than a decade later, the number of publications focused on improvement of teaching has not waned and researchers continue to document instructors’ delivery of subject matter (Stevenson and Harris, 2014), students’ learning styles (Romanelli et al., 2009), and the best practices to align teaching and learning styles (Dinçol et al., 2011). Across disciplines, there is little debate that mastery of the subject matter is critical (Peer, 2015; Ford, 2016; Keeley et al., 2016; Noll, 2017), but it is also widely accepted that expertise and experience do not always equate to being a quality teacher (Hattie, 2003).

Animal science is a unique discipline, as its educational outcomes innately align with Bloom’s Taxonomy (1956) of learning objectives (e.g., understanding and application), and Kolb’s Learning Style (1984) that promotes experiential learning and problem-solving. Despite an increase in public and societal recognition of outstanding teachers in recent years, there is a disproportionately small number of teachers representing the animal sciences. There is a multitude of challenges for educators, especially those beginning their careers (Mundt and Connors, 1999). If higher educational institutions continue to honor their commitment to produce quality animal scientists, these early-career educators need the tools to emulate the top animal science teachers. There are animal scientists who are considered excellent teachers (Buchanan, 2008), but there is still a gap between the number of examples of excellent teachers in other disciplines (e.g., psychology, criminal justice, education) compared with animal science, suggesting a need to identify and describe the characteristics of top animal science teachers. Our objectives were to observe and quantify the traits portrayed by top animal science educators and their classrooms of students. We hypothesized that, despite a diverse background among award-winning animal science teachers, common themes and challenges would emerge in addition to a relatively uniform classroom learning experience.

## MATERIALS AND METHODS

Data collection methods were deemed exempt by the University of Findlay Institutional Review Board. The inclusion criterion for animal science teachers was that they must have been bestowed excellence in teaching award through the American Society of Animal Science, the North American Colleges and Teachers of Agriculture, or the National Institute of Food and Agriculture (USDA) since 1994. Teachers meeting the criteria (*n* = 21) were contacted up to four times over a 4-mo period to determine interest and willingness to participate in the study. The teachers participating in the study (*n* = 4) were able to choose the class and day for observation (Table 1), and they responded to a series of questions prior to the observed lecture (Table 2).

**Table 1.** Description of observed classes taught by award-winning animal science professors

Course	Topic	Catalog level	Student level	Impetus	Prerequisite
Agriculture Biochemistry	Citric acid cycle	251	Sophomore	Required	Chemistry
Applied Livestock Genetics	Sire selection	4323	Junior	Required	Genetics
Physiology of Reproduction	Placentation and parturition	4314	Junior	Required	Anatomy and Physiology
Sheep Science	Diseases	229	Sophomore	Elective	Introduction to Animal Science

**Table 2.** Questions asked of award-winning animal science teachers before observation of their teaching

Question
How long have you been teaching?
Why did you become a teacher?
How would your students describe you?
What is the biggest challenge you face?

Each lecture was captured in its entirety using a digital video camera (Hero3+, GoPro, Inc., San Mateo, CA, USA) and a digital all-inclusive camera (Samsung Gear 360, Samsung Electronics Co., Ltd., Seoul, South Korea). Actions, pedagogical trends, and instructor–student interactions were quantified by a single observer through play-back technology. Initially, based on the survey responses by the teachers, instances of humor, rapport, demand were noted during the play-back. When an event was visualized by the observer, it was recorded as such and the total occurrences of each event were calculated. Events included, but were not limited to, specific student distractions, questioning, teaching styles, movements, and communicating. Recordings of pedagogical instances for professional development use are available upon request.

## RESULTS AND DISCUSSION

The aim of this study was to evaluate what top animal science teachers did in their classrooms and the responses they received from their students in order to provide guidance and recommendations for those wishing to improve their teaching. Although there are many ideas of what makes a great teacher, few are based on direct observation of classroom performance and even less that consider the animal science discipline. There are arguably three excellent summaries of teaching in the animal sciences (Washburn, 1958; Taylor and Kauffman, 1983; Buchanan, 2008) that have helped shaped animal science educators; however, the most recent was published more than 12 yr ago. It would be difficult to identify any animal scientist who would be supportive of having research findings in their area of interest published only every 10–12 yr, so considering the often-cited three-part function of the animal science discipline (teaching, research, extension), how can a lack of publications on teaching be considered acceptable? Additionally, the previous summaries focused on content, curricula, and evaluation, and not specifically on what best teachers do. Therefore, this study bridges the gap between what should be taught in an animal science curriculum to meet stakeholder needs and how top teachers accomplish that.

Teacher responses to the prelecture questions indicated that the average length of time teaching was 32.3 ± 3.9 yr. Teachers chose this specific profession because: 1) it was the pathway to have a successful career aligned with their life’s ambitions, 2) it was the best way to combine research and teaching, and 3) the satisfaction they derived from interacting with students and explaining a variety of topics. These responses coincide with the top reasons that college students became teachers historically, namely a desire to help young people and the ability to continue intellectual growth (Jantzen, 1981). Interestingly, student perception of teaching quality and performance in the science disciplines varies with the age of the teacher (Kinney and Smith, 1992): younger teachers (less than 35 yr) and older teachers (greater than 55 yr) had lower evaluations than those teachers between 35 and 55 yr old. The teachers observed in the current study fell into this age range of high-quality teaching and performance levels when considering the year the teaching recognition was received.

The responses to how the teachers believed their students would describe them are listed in alphabetical order in Table 3. Responses can easily be sorted and placed into three distinct descriptor categories: humorous (aloof, funny), rapport (approachable, concerned, empathetic, engaged, helpful, understanding), and demanding (inflexible, intimidating, rigid). It should not be surprising that top animal science teachers are described within these three categories, as there is extensive evidence that all three contribute to improvements in student learning.

**Table 3.** Self-reported answers by award-winning animal science teachers for how students would describe them. Answers are listed alphabetically

How would your students describe you?
Aloof
Approachable
Concerned
Demanding
Empathetic
Engaged
Funny
Helpful
Inflexible
Intimidating
Rigid
Understanding

Professors that use humor in the classroom as a pedagogical technique increase their credibility, likability, image, and overall teaching effectiveness (Deiter, 2000). When there is an increase in teaching effectiveness, students typically improve academically, and it could be concluded that using humor in the classroom can improve the retention and recollection of the material by the students (Garner, 2006). Previous research has also shown that the use of humor while teaching positively influences and improves the average student engagement levels with the professor and within the class (Nienaber et al., 2019). An increase in student engagement during class (due to humor) creates more open communication and thus contributes to the second category described, rapport (Darling and Civikly, 1986).

Early on, it was evident that the nature of the animal science discipline required a strong rapport between students and teacher, otherwise high-quality critical thinking and experiential learning would not take place (Kauffman et al., 1984). The establishment of a positive student/instructor rapport increases student motivation to learn and succeed (Chickering and Gamson, 1987) while at the same time increasing the students’ expectations for success and reaching their goals (Estepp and Roberts, 2015). The positive learning environment that can be created by instructor/student rapport (Wilson et al., 2010) is influenced by the teacher’s verbal and nonverbal involvement (Pintrich and Zusho, 2007). This could not be more evident in the animal sciences because animals do not speak a human language and multiple aspects of animal husbandry are accomplished nonverbally.

The final descriptor category (teachers are demanding) could be considered in pedagogical terms as teachers having high expectations of students. Expecting more from students typically results in getting greater results from students. These higher expectations do not equate to assigning more work or making the examinations more difficult, but instead translate into asking students to perform as best as they can. When teachers expect more from students, teachers, and institutions tend to expect more from themselves (Chickering and Gamson, 1987). When teachers have high expectations for their students, they likely teach at a higher level and create a more positive learning atmosphere, leading to higher student achievement (Goldenberg, 1992; Jenkins, 2016).

Animal science teachers who include humor in their teaching style and build rapport with their students while concurrently holding high expectations for success, create a memorable and positive influence on their students, contributing to the justification of them being recognized as outstanding educators.

The responses to what the teachers believed the biggest challenges they currently face in the classroom are listed in Table 4. Three themes emerge from the responses given: engagement (effort from the students, use of electronics, student engagement), connection (disconnect between faculty and students, students from more diverse backgrounds, students with little agriculture and/or science background), and application (loss of the big picture). A final challenge mentioned was that the average student in the classroom has more nonacademic distractions than previous generations of students. While this could be accurate, it is beyond the scope of this study and was not considered for further discussion.

**Table 4.** Self-reported answers by award-winning animal science teachers for the biggest challenges they face. Answers are listed alphabetically

What is the biggest challenge you face?
Disconnect between faculty and students
Electronics
Lack of effort from students Lack of student engagement Loss of the big picture (only a box to check to earn a degree)
Student population comes from more diverse backgrounds
Students have less agriculture and science background
Students have more nonacademic distractions in their lives

The three categories that emerged could describe the challenges faced in almost any classroom of higher education, including most animal science classrooms. What makes these teachers stand out as exemplary animal science teachers is how they approached and addressed each of these challenges in their classroom on a daily basis (Table 5). It is no surprise that any individual who can engage and connect with students while making the information applicable would be considered one of the top-ranking college professors (Slate et al., 2009).

**Table 5.** Events occurring in all classrooms of award-winning animal science teachers, independent of each other. Answers are listed alphabetically

Common themes emerging
Called on students by their name
Lecture was not continuous/uninterrupted
Previous material was reviewed at the beginning of lecture
Teachers directly asked the students questions and expected immediate answers/feedback
Teachers distributed something to the students during the lecture
Used multiple forms of course content delivery during lecture

Personal traits of the teachers are influential to the student perceptions of their instructors (Wang et al., 2007; Kadioglu Ates and Kadioglu, 2018). Teachers that connect and engage their classrooms have more involved students, enhance quality lecture experiences, and ultimately attain higher levels of student performance and learning success. Irrespective of discipline or academic level, high-quality teachers are continually identified as being able to engage students in the learning process (Acker, 2003; Benekos, 2016). Engaging the students in the classroom facilitates a platform for open, two-way communication (Acker, 2003) which generates active or experiential learning and questioning (Guskey and Easton, 1983). In agreement with these previous findings, top animal science teachers engaged and connected to their classes by calling their students by name, asking directed questions to specific students, reviewing material, and providing multiple platforms through which the material was delivered during the class. Increasing the modality of content delivery within a single class provides more opportunities for active learning, which promotes a more cohesive knowledge base and understanding of the material (Gardner and Belland, 2012).

The animal science teachers also met the challenge of teacher–student disconnect and student apathy by taking the time and opportunity to relate concepts to tangible constructs the students could understand. Making the course content applicable to students’ lives is essential, given the diverse backgrounds that comprise a college classroom. Including more active learning strategies and making the content relevant to students’ lives improves a professor’s effectiveness (Aulls, 2004). By connecting knowledge to the students, the best teachers further engage students in the learning process and improve the quality of education (Onwuegbuzie et al., 2007).

Teaching has historically been an important and integral part of the animal sciences (Buchanan, 2008), although perhaps more difficult to quantify and describe than research or extension. This study aimed to evaluate the common themes that emerged among those animal science teachers considered to be the best. Our results suggest that great teachers have an exceptional grasp of their subject matter, understand their audience, and easily identify the challenges they face. Our results demonstrate that, although everyone teaches differently and could have a unique set of challenges, the best teachers do not divert away from the challenges, but rather directly face and attempt to overcome them. The best animal science teachers value student learning as paramount to their careers, and animal science teachers everywhere can apply in their own environment what these award-winning teachers do in order to foster improved student learning.
